# Brevilin A shows an anti-tumor role in prostate cancer via the lncRNA H19/miR-194/E2F3 signaling pathway

**DOI:** 10.18632/aging.204744

**Published:** 2023-05-30

**Authors:** Pinghong You, Liangyou Tang, Yanjie Zhu, Yuchang Tian

**Affiliations:** 1 Department of Urology, People’s Hospital of Deyang City, Deyang 618000, Sichuan, China

**Keywords:** Brevilin A, prostate cancer, signaling pathway, lncRNA H19

## Abstract

Brevilin A, a natural sesquiterpene lactone extracted from *Centipeda minima*, has been found with antitumor properties. Our study probed the functions of Brevilin A in prostate cancer cells and the mechanisms among Brevilin A, lncRNA H19, miR-194, and E2F3 on the biological behaviors of the cells. CCK8, Transwell, and TUNEL staining assays examined the impact of Brevilin A on prostate cancer cell proliferation, migration, invasion, and apoptosis, respectively. qRT-PCR and western blot determined lncRNA H19, miR-194, and E2F3 profiles. The influence of Brevilin A on the profiles of lncRNA H19, miR-194, and E2F3 was measured. A xenograft model of prostate cancer nude mice was taken to confirm the impact of Brevilin A and lncRNA H19 on cancer cell growth. Consequently, Brevilin A dampened prostate cancer cell proliferation, migration, and invasion, suppressed the expressions of lncRNA H19 and E2F3, and enhanced miR-194 level. LncRNA H19 and E2F3 were uplifted, whereas miR-194 was abated in prostate cancer cells and tissues. LncRNA H19 targeted miR-194 to positively modulate E2F3 expression, boosted DU145 cell proliferation, invasion, and migration, and curbed apoptosis. In the xenograft model, Brevilin A repressed tumor growth, whereas lncRNA H19 fostered tumor growth. Brevilin A suppressed the promotive effect of lncRNA H19 in PC cell growth *in vivo*. To conclude, Brevilin A modulates the biological behaviors of prostate cancer cells via the lncRNA H19/miR-194/E2F3 axis. Brevilin A exerts an anti-tumor function in prostate cancer.

## INTRODUCTION

Prostate cancer (PCa) is one of the most prevalent types of cancer in older men and seriously threatens human health [1]. The initiation and development of PCa have been confirmed to be associated with the activation of oncogene and inactivation of tumor suppressors [2]. The main challenges of PCa management include early diagnosis and treatment due to its high heterogeneity [3]. Therefore, investigating the pathogenesis of prostate cancer is critical to identifying new therapeutic targets for effective therapies.

Brevilin A, a sesquiterpene lactone derived from *Centipeda minima* [4], has been demonstrated to exert an anti-tumor function in many cancers. For instance, Brevilin A, an effective anti-tumor bioactive molecule, can elicit apoptosis in U87 glioblastoma cells [5]. When it comes to lung cancer, Brevilin A can suppress proliferation in non-small cell lung cancer cells and induce their morphological alterations [6]. Moreover, Brevilin A induces nasopharyngeal carcinoma cell cycle arrest, suppresses the profile of Bcl-2, and enhances apoptosis [7]. These studies have revealed that Brevilin A boasts a potential anti-tumor effect in PCa.

E2F Transcription Factor 3 (E2F3), a member of the E2F family, determines the transition time of cell G1/S phase, DNA synthesis rate, and cell proliferation [8]. The excessive expressions of E2F3 can enhance cell proliferation as there is not enough Rb to bind all E2F3 family members [9], and a number of studies have indicated that E2F3 is overexpressed in lung cancer [10], ovarian cancer [11], and prostate cancer [12], suggesting the abnormal profile of E2F3 is inextricably correlated with tumorigenesis [13].

Micro-RNAs (miRNAs) are endogenous, small, non-coding molecules [14]. They can modulate many biological behaviors like tumor differentiation, proliferation, and apoptosis [15]. miRNAs including miR-148-3p, miR-32, and miR-210-3p are over-expressed in prostate cancer [16, 17], while miR-200, miR-29b, miR-205, and miR-940 are down-regulated [18], suggesting that the aberrant profiles of miRNAs closely pertain to prostate cancer development. As another group of non-coding RNAs, long non-coding RNAs (lncRNAs) have over 200 nucleotides in length, possess conserved secondary structures, and interact with proteins, DNA, and RNAs to regulate gene expression at transcription and post-transcription levels [19]. Some reports have substantiated that lncRNAs can drive cancer phenotypes, such as regulating tumor cell proliferation and apoptosis and shortening or prolonging the cell cycle [20]. Some lncRNAs can play an inhibitory role in prostate cancer development [21]. Studies have corroborated the functions of H19 single-nucleotide polymorphisms (SNPs) in prostate cancer by affecting perineural invasion of this cancer [22]. Our research intended to probe the regulatory relationship among Brevilin A, lncRNA H19, miR-194, and E2F3 and their influence on the biological behaviors of prostate cancer cells, and the mechanism of Brevilin A affecting lncRNA H19 expression in prostate cancer was investigated.

## MATERIALS AND METHODS

### Clinical samples

Tumor and adjacent normal tissue samples of 56 patients with prostate cancer were collected, and no patients underwent chemotherapy prior to surgery. All fresh specimens were quickly cryopreserved in liquid nitrogen, and confirmed by pathological diagnosis. The collection of specimens was approved by the Ethics Committee of the People’s Hospital of Deyang City. The informed consent was also signed by patients or their family members.

### Cell culture and transfection

The prostate epithelial cell line PrEC and prostate cancer cell lines (PC3, LNCap, DU145) were cultured in DMEM adding with 10% fetal bovine serum (FBS) (Gibco, CA, USA), 100 U/ml penicillin and 100 μg/ml streptomycin (Beyotime, Shanghai, China) with 95% air and 5% CO_2_ at 37°C. About 24 hours prior to transfection, cells in good conditions were seeded in 6-well plates (5 × 10^5^ cell/well). As the cells grew to 70%–90% confluence, transfection was conducted as per the instructions of Lipofectamine™ 2000 (Invitrogen, CA, USA).

### qRT-PCR

TRIzol (Invitrogen, CA, USA) was adopted to separate RNAs from the cells or tissues, and the RNA concentration and integrity were examined through UV spectrophotometry and agarose gel electrophoresis, respectively. All RNAs were synthesized into cDNAs using the reverse transcription kit (Thermo Fisher, CA, USA). PCR was performed in line with the protocol of the qRT-PCR kit (Thermo Fisher, CA, USA). The reaction system was set as the following: 30 seconds’ pre-denaturation at 94°C; 5 seconds’ denaturation at 94°C, 15 seconds’ annealing at 60°C, 10 seconds’ extension at 72°C, and 45 cycles of amplification. lncRNA H19 and miR-194 expressions were normalized by U6, and the mRNA profile of E2F3 was corrected by GAPDH. The outcomes were presented as 2^−ΔΔCT^. Primer sequences are detailed below:

lncRNA H19-F: 5′-ATCGGTGCCTCAGCGTTCGG-3′; lncRNA H19-R: 5′-CTGTCCTCGCCGTCACACCG-3′; miR-194-F: 5′-ATGGACCTGGGGCCAGCGAAG-3′; miR-194-R: 5′-TCTGGCCTGGGAGCGTCG-3′; E2F3-F: 5′-AGAAAGCGGTCATCAGTACCT-3′; E2F3-R: 5′-TGGACTTCGTAGTGCAGCTCT-3′; U6-F: 5′-CTCGCTTCGGCAGCACA-3′; U6-R: 5′-AACGCTTCACGAATTTGCGT-3′; GAPDH-F: 5′-GTCAACGGATTTGGTCTGTATT-3′; GAPDH-R: 5′-AGTCTTCTGGGTGGCAGTGAT-3′.

### Western blot

The total protein was extracted using RIPA lysis buffer (Beyotime, Shanghai, China), quantified with BCA, and denatured after the addition of a buffer solution. With 50 μg of the protein administered to each lane, the samples were subjected to isolation through 12% SDS-PAGE and then moved onto PVDF membranes. After being sealed with 5% skim milk at 37°C for an hour, the membranes were incubated along with primary antibodies Anti-E2F3 (Abcam, 1:500, ab152126, MA, USA) and Anti-GAPDH (Abcam, 1:1000, ab181602, MA, USA) at 4°C overnight. GAPDH was taken as the internal parameter. PBS was employed to flush the membranes three times, which were then incubated with the Horse Radish Peroxidase (HRP)-labeled anti-rabbit secondary antibody (1:1000) (Abcam, ab6721, 1:2000, MA, USA) for 60 minutes at room temperature (RT). The membranes were rinsed with TBST three times. The ECL kit was utilized to visualize protein bands.

### Immunohistochemistry

Paraffin sections were deparaffinized and rehydrated after being placed in an oven overnight at 65ºC. 3% H_2_O_2_ solution was administered to incubate the slices for 15 minutes at RT to block endogenous peroxidase activities. The sections were kept in citrate buffer and microwaved for antigen repair. 5% normal goat serum was given to block and incubate the sections for 15 minutes at RT. Primary anti-E2F3 antibody (1:100) (Proteintech, Wuhan, China) or Ki67 antibody (1:100) (Abcam, ab15580, MA, USA) was added for maintaining at 4°C overnight. The Horse Radish Peroxidase-labeled anti-rabbit secondary antibody (1:200) (Abcam, ab6721, MA, USA) was administered for 30 minutes’ incubation at RT. The streptavidin working solution labeled with the horseradish enzyme was given for 30 minutes’ incubation at 37°C. After color development, haematoxylin was adopted for staining at RT for 2 minutes, followed by dehydration and blocking with neutral resins. The sections were examined with an upright microscope (Olympus, Japan).

### Dual-luciferase reporter gene assay

TargetScan forecast the binding site between lncRNA H19 and miR-194 as well as the binding site between miR-194 and E2F3. All luciferase reporter vectors (pMIR-H19-wt, pMIR-H19-Mut, pMIR-E2F3-wt, and pMIR-E2F3-Mut) were established by Promega (WI, USA). DU145 cells, inoculated onto 96-well plates, were cultured to 70% confluence. Lipofectamine^®^ 2000 (Invitrogen; Thermo Fisher Scientific, Inc., CA, USA) was employed to transfect pMIR-H19-wt, pMIR-H19-Mut, pMIR-E2F3-wt, pMIR-E2F3-Mut, and miR-194 mimics or negative controls into DU145 cells. Following 48 hours’ transfection, dual luciferase activity was gauged as per the supplier’s instructions. All experiments were conducted in triplicate and duplicated three times.

### CCK8 assay

To measure the cytotoxicity and anti-proliferation effect, we cultured PC3, LNCap, and DU145 cells in Brevilin A (Cat.No. HY-N2959, MedChemExpress, NJ, USA) of different concentrations (0, 5, 10, 20, 40, 80 μM) for 24 hours. Then, the cells were seeded onto 96-well plates, with 200 μL of cell suspension in each well (density: 1 × 10^4^cells/well). Subsequent to 24 hours’ routine culture, 10 μL CCK-8 solution was given to each well as per the instructions of the CCK-8 kit (Beyotime, Shanghai, China). After the cells were incubated for 1–2 hours at 37°C, a microplate reader (BioTek, WA, USA) was adopted to gauge the absorbance value at 450 nm.

### Transwell assay

In cell invasion assay, Matrigel (Invitrogen, CA, USA) was maintained overnight at 4°C and liquefied with a dilution of 1:6 with a medium. In cell migration assay, Matrigel was not used. The three PCa cells (PC3, LNCap, DU145) were seeded on uncoated plates for migration assays. 100 μL cell suspension and 600 μL 10% serum medium (Thermo Fisher Fisher, CA, USA) were administered to the upper and lower chambers, respectively, and the cells were cultured in an incubator at 37ºC at 5% CO_2_ for 24 hours. After non-migratory cells were wiped off, the chambers were fixed in 95% ethanol for 5 minutes and stained in 0.5% crystal violet (Beyotime, Shanghai, China) for 10 minutes. The cells in the lower chamber were observed under a microscope.

### TUNEL staining

One Step TUNEL Apoptosis Assay Kit (Cat.No. C1086, Beyotime, Shanghai, China) was used for evaluating cell apoptosis. The three PCa cells (PC3, LNCap, DU145) were dealt with Brevilin A for 24 hours, then they were immobilized with 4% paraformaldehyde for 30–60 minutes, flushed twice with PBS. The cells received penetration by 0.3% Triton X-100, flushed twice with PBS, and incubated with 50 μL TUNEL working solution was added to incubate the cells for 30 minutes at 37°C. After washing with PBS three times, the nucleus was stained with DAPI (Beyotime, Shanghai, China). Finally, the fluorescence signals were observed using a fluorescence microscope (Olympus, Japan).

### The establishment of the nude mouse xenograft model of prostate cancer

Fifty BALB/c nude mice, 6 weeks of age and 14–17 g in weight, were randomized to the control group, Brevilin A group, lncRNA H19 group, the lncRNA H19 + Brevilin A group, and the negative control group (five mice/group). Brevilin A (10 mg/kg, 20 mg/kg) was given by intraperitoneal injection once a day from the second week after tumor cell injection [23]. All nude mice were reared under SPF housing with fresh food and water. After transfection, the concentration of DU145 cells was adjusted to 1 × 10^7^ cells/ml, and 0.2 ml was injected into each mouse with a 1 ml syringe. The tumor volume was gauged every 7 days. Twenty-eight days later, high-concentration CO_2_ was adopted to euthanize the animals. Their tumor tissues were excised, weighed, and immobilized with formaldehyde. This study has been approved by the Ethics Committee of People’s Hospital of Deyang City. We made all attempts to minimize suffering of experiment animals and limit the number of animals used.

### Statistical analysis

The SPSS Statistics 22.0 software was introduced for statistical analysis. Data were presented as means ± SD. Statistical comparison was performed via Student’s *T*-Test between two different groups and through one-way ANOVA among three or more groups followed by Tukey’s post hoc test. A *p*-value less than 0.05 was considered statistically significant.

### Data availability statement

The data sets used and analyzed during the current study are available from the corresponding author on reasonable request.

## RESULTS

### Brevilin A suppressed prostate cancer cell proliferation and growth

**Figure 1:**
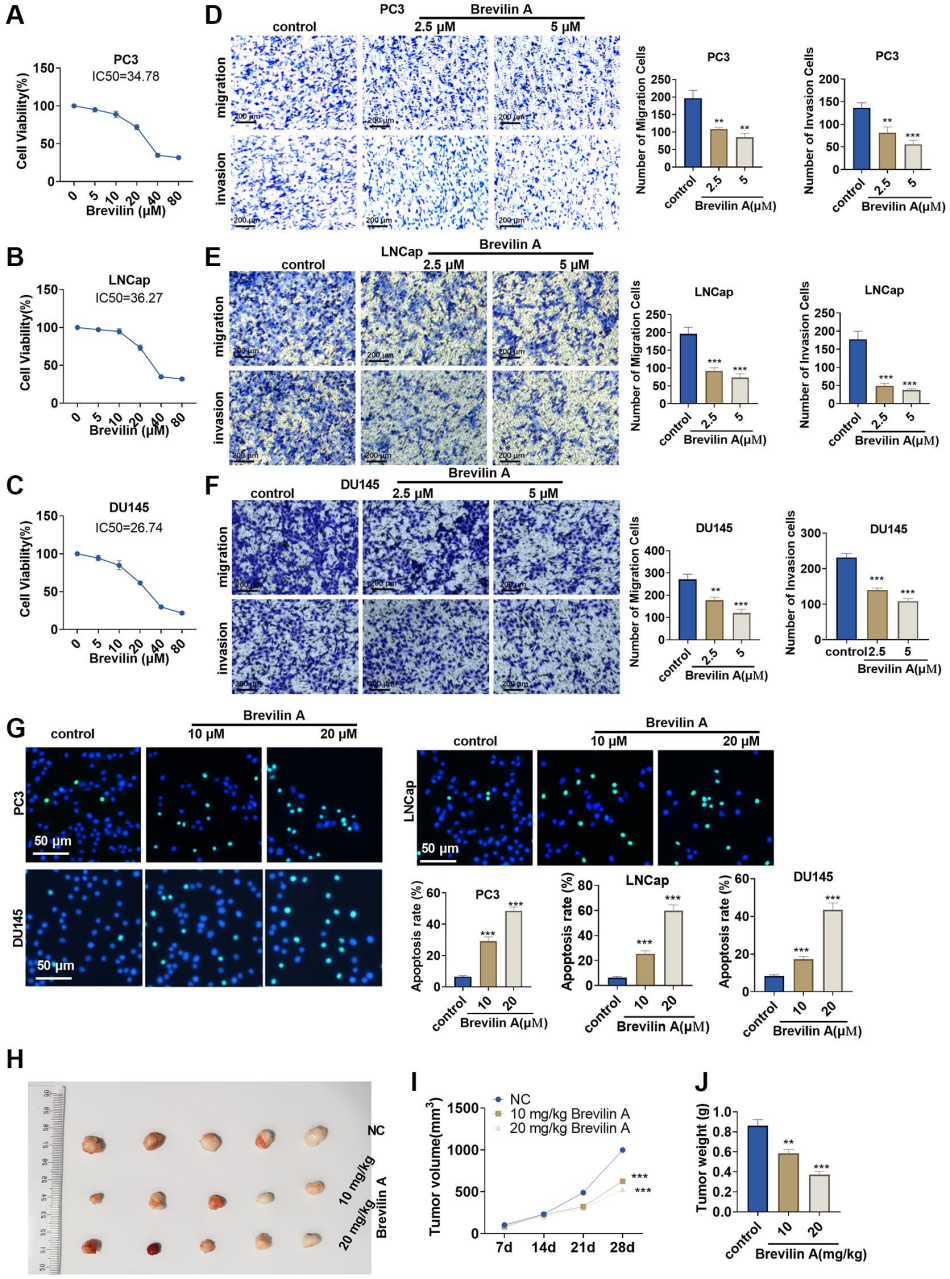
**Brevilin A dampened prostate cancer cell proliferation.** Brevilin A of different concentrations (0–80 μM) was adopted to treat prostate cancer cells (PC3, LNCap, DU145). (**A**–**C**) CCK8 assay examined cell viability. (**D**–**F**) Transwell checked cell migration and invasion. (**G**) TUNEL assay monitored apoptosis of PC3, LNCap, DU145 cells. (**H**) DU145 cells were taken to construct a nude mouse xenograft model of prostate cancer, and Brevilin A (10 mg/kg, 20 mg/kg) was administered for treatment. The tumors were excised 28 days later. (**I**, **J**) Tumor volume and tumor weight were calculated every 7 days. ns *P* > 0.05, ^*^P < 0.05, ^**^P < 0.01, ^***^P < 0.001 (vs. control).

To confirm the influence of Brevilin A on prostate cancer, we implemented CCK8 to track alterations in the viability of PC3, LNCap, and DU145 cells treated with Brevilin A of different concentrations. It emerged that the higher the concentration of Brevilin A was, the weaker the viability became (Figure 1A–1C). As per our calculation, the IC_50_ values of Brevilin A against PC3, LNCap, and DU145 cells were 34.78, 36.27, and 26.74 μM, respectively. To probe the impact of Brevilin A on PCa cell migration and invasion, we exposed the cells to 2.5 and 5 μM Brevilin A for treatment and then carried out Transwell assay. As a result, in contrast with the control group, Brevilin A attenuated invasion and migration (Figure 1D–1F). As displayed by TUNEL staining, by contrast to the control group, Brevilin A boosted cell apoptosis (Figure 1G). To further confirm the antitumor effects of Brevilin A, we conducted *in-vivo* assays. The data showed that Brevilin A obviously attenuated cell growth (Figure 1H–1J). These data revealed that Brevilin A weakened PCa cell viability, migration, invasion and elicited apoptosis.

### The impact of Brevilin A on lncRNA H19, miR-194, and E2F3 expressions

**Figure 2:**
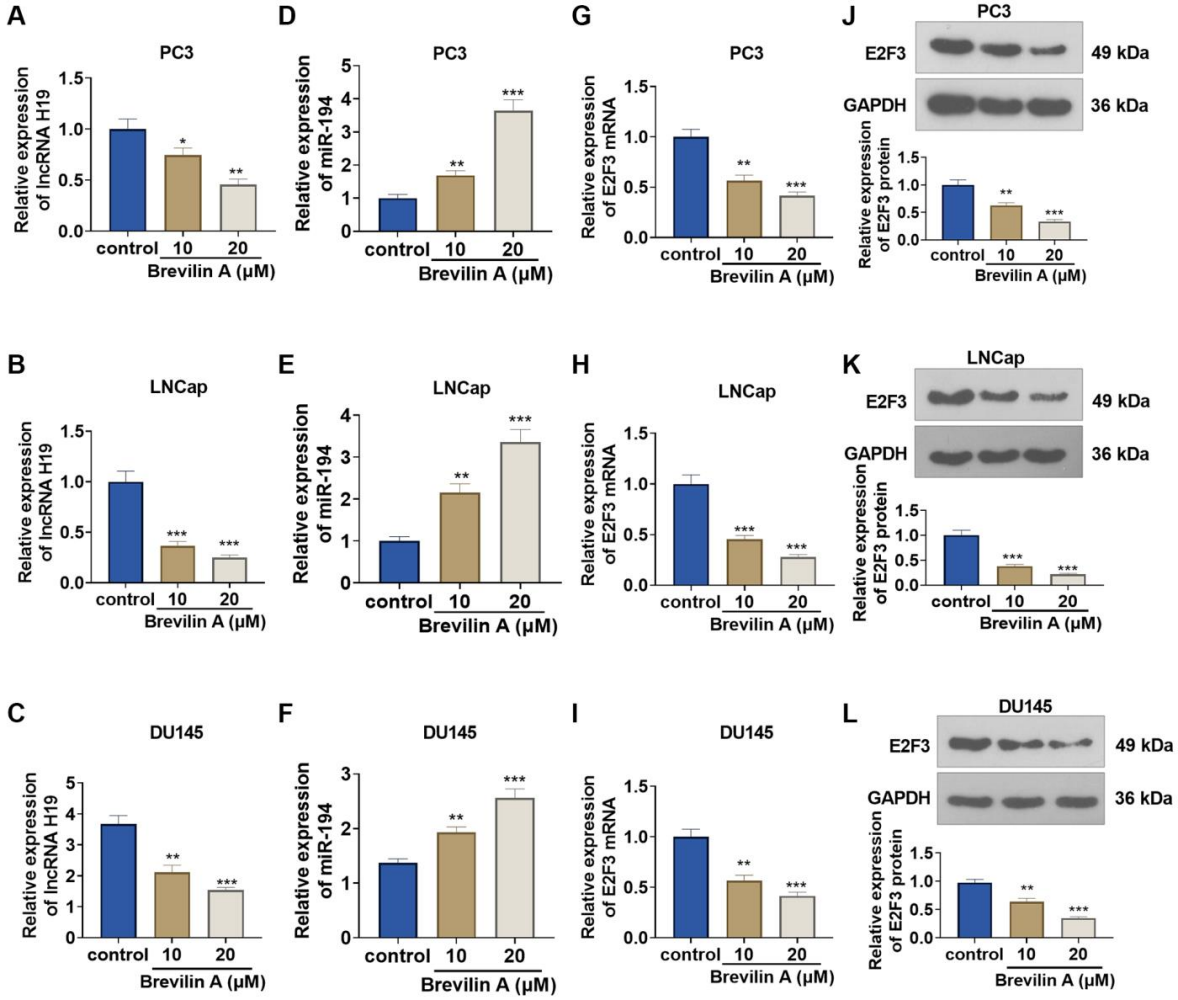
**The impact of Brevilin A on lncRNA H19, miR-194, and E2F3 expressions.** DU145 cells were treated at the concentrations of 10 μM and 20 μM, which were calculated through the IC_50_ values of Brevilin A. (**A**–**I**) qRT-PCR determined lncRNA H19 (**A**–**C**), miR-194 (**D**–**F**) and E2F3 mRNA (**G**–**I**) expressions. (**J**–**L**) Western blot measured E2F3 expression. ^*^P < 0.05, ^**^P < 0.01, ^***^P < 0.001 (vs. control), *n* = 3.

To corroborate the impact of Brevilin A on the profiles of lncRNA H19, miR-194, and E2F3, we cultured DU145 cells in 10 and 20 μM Brevilin A for 24 hours. qRT-PCR confirmed lncRNA H19 and miR-194 expressions, indicating that in contrast with the control group, lncRNA H19 expression declined, while miR-194 expression rose (Figure 2A–2F). qRT-PCR and Western blot denoted that by contrast to the control group, E2F3 mRNA and protein expression was abated following Brevilin A administration (Figure 2G–2L). These findings demonstrated that Brevilin A repressed lncRNA H19 and E2F3 expressions and enhanced miR-194 expression in the PCa cells.

### lncRNA H19, miR-194, and E2F3 expressions in prostate cancer cells and tissues

**Figure 3:**
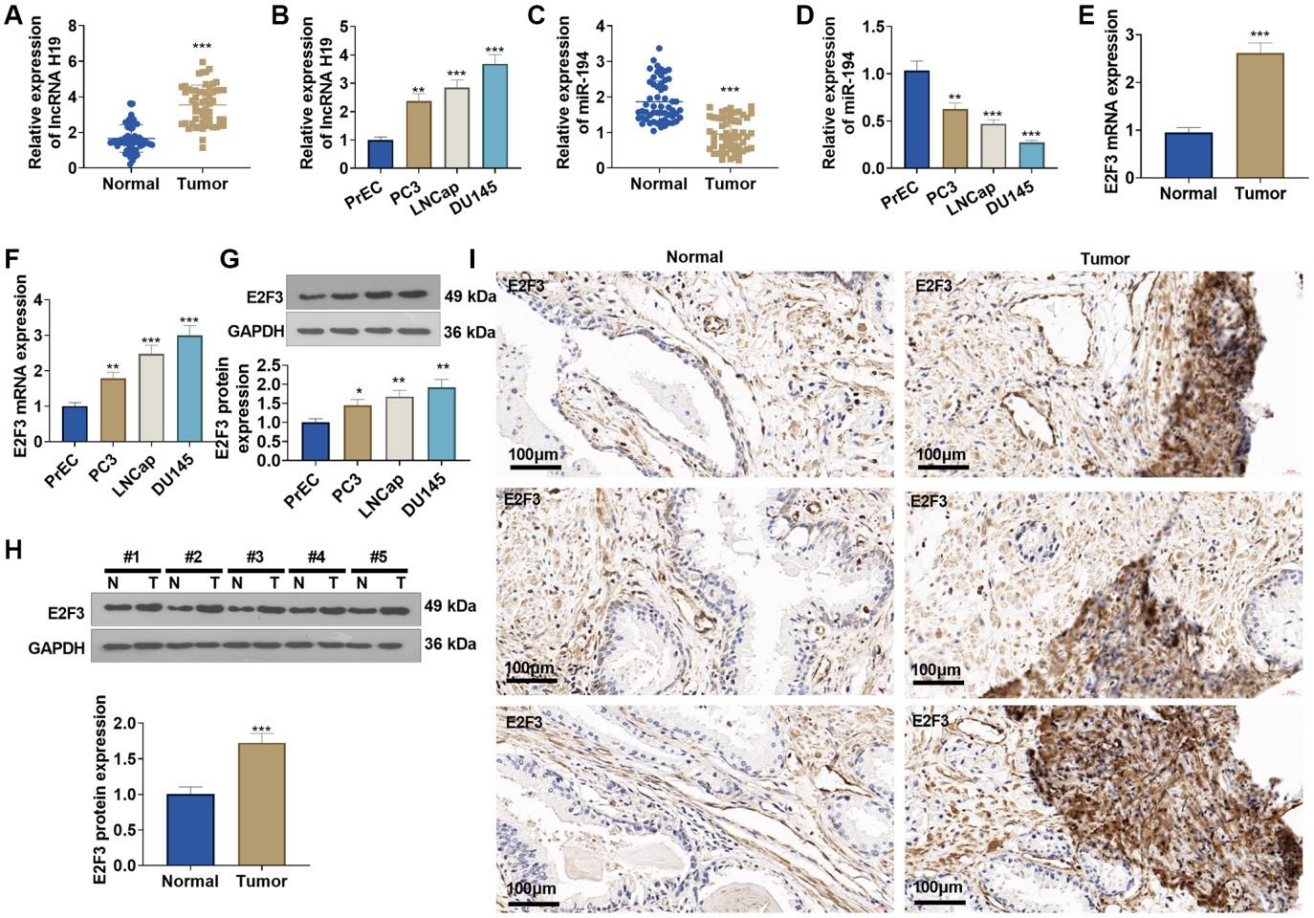
**The profiles of lncRNA H19, miR-194, and E2F3 in prostate cancer tissues and cell lines.** (**A**, **B**) qRT-PCR detected lncRNA H19 expression in prostate cancer tissues and cell lines. (**C**, **D**) qRT-PCR determined miR-194 expression in prostate cancer tissues and cell lines. (**E**, **F**) qRT-PCR examined E2F3 mRNA expression in prostate cancer tissues and cell lines. (**G**, **H**) Western blot was performed for assaying E2F3 protein level. (**I**) Immunohistochemistry checked E2F3 expression in prostate cancer tissues. Scale bar = 50 μm. ^*^P < 0.05, ^**^P < 0.01, ^***^P < 0.001 (vs. Normal or PrEC), *n* = 3.

qRT-PCR suggested that lncRNA H19 and E2F3 expressions were obviously elevated in prostate cancer tissues versus adjacent normal tissues (Figure 3A, 3C, 3E), while miR-194 expression was substantially lowered (Figure 3B). Western blot and immunohistochemistry reflected that E2F3 was highly expressed in prostate cancer tissues as opposed to adjacent normal tissues (Figure 3H, 3I). Additionally, lncRNA H19 and E2F3 were more highly expressed in prostate cancer cell lines than in human prostate epithelial cells PrEC (Figure 3B, 3F, 3G), whereas miR-194 expression exhibited the opposite result (Figure 3D). All findings confirmed that the aberrant profiles of lncRNA H19, miR-194, and E2F3 might be correlated with prostate cancer.

### Interaction among lncRNA H19, miR-194, and E2F3

**Figure 4:**
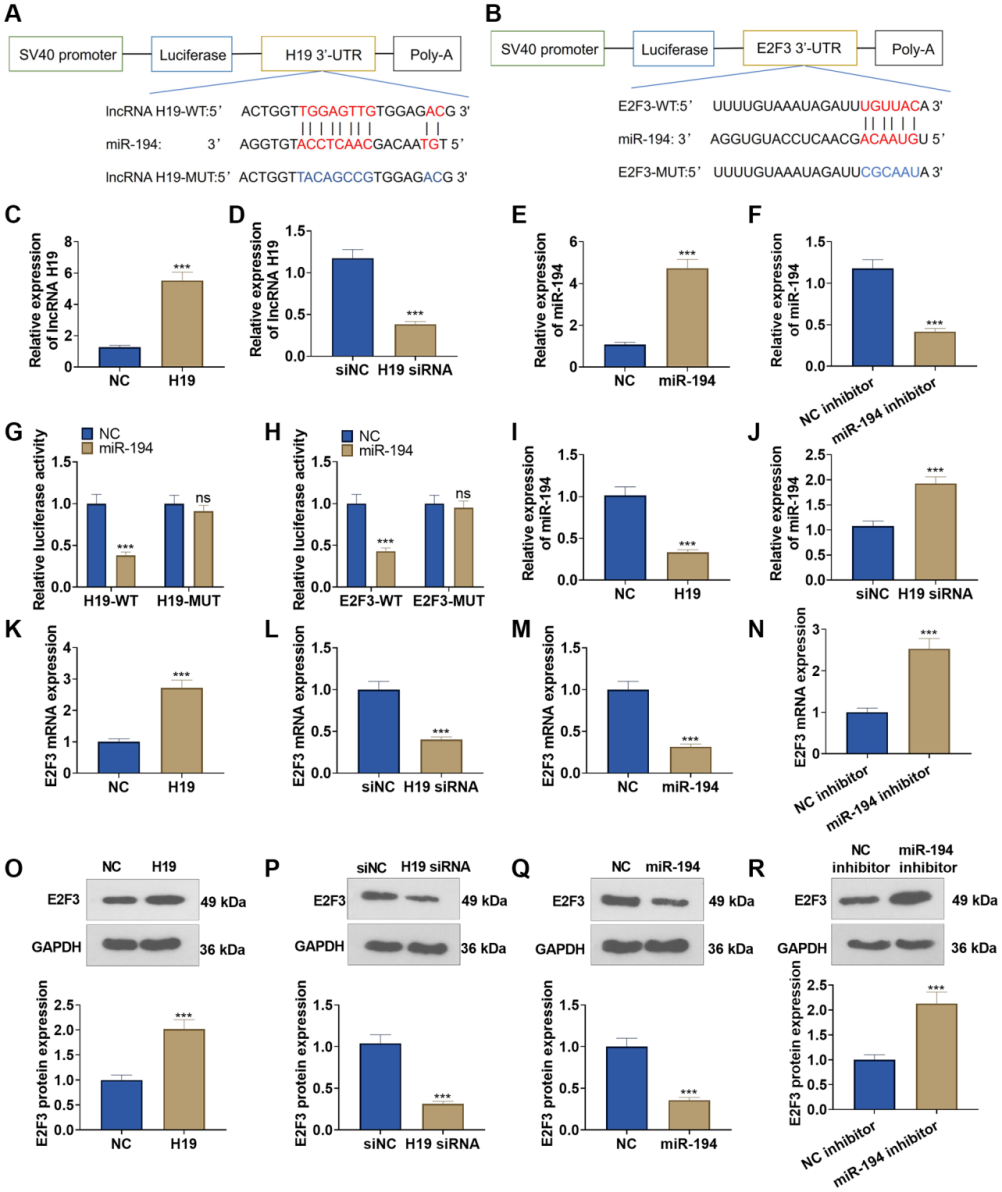
**The interaction among lncRNA H19, miR-194, and E2F3.** (**A**, **B**) Starbase database the binding site between lncRNA H19 and miR-194 as well as the binding site between miR-194 and E2F3. (**C**–**F**) qRT-PCR examined the profiles of lncRNA H19 and miR-194. (**G**, **H**) Dual luciferase reporter gene assay measured luciferase activity of DU145 cells. (**I**, **J**) The miR-194 level was probed via qRT-PCR. (**K**–**R**) Western blot and qRT-PCR tested the effects of lncRNA H19 and miR-194 on E2F3 expression. ns *P* > 0.05, ^***^P < 0.001, *n* = 3.

The TargetScan database indicated that lncRNA H19 could bind to miR-194, and miR-194 could combine with E2F3 (Figure 4A, 4B). qRT-PCR unraveled that after lncRNA H19 overexpression plasmids were transfected into DU145 cells, lncRNA H19 expression was greatly heightened, and lncRNA H19 expression was lowered after the transfection with lncRNA H19 siRNA (Figure 4C, 4D). Transfecting miR-194 mimics could drive up miR-194 expression in DU145 cells, but transfecting the miR-194 inhibitor could result in a lower expression of miR-194 (Figure 4E, 4F), which signaled the success in transfection. Dual luciferase activity assay unveiled that miR-194 repressed the luciferase activities of lncRNA H19-WT and E2F3-WT but exerted no impact on lncRNA H19-MUT and E2F3-MUT (Figure 4G, 4H), which revealed that miR-194 could serve as the target miRNA of lncRNA H19, and miR-194 could specifically combine with the 3′-UTR of E2F3. We also examined the profile of miR-194 when lncRNA H19 was overexpressed and knocked down. Upon lncRNA H19 overexpression, miR-194 expression was lowered. As lncRNA H19 was knocked down, miR-194 expression was uplifted (Figure 4I, 4J). Western blot and qRT-PCR reflected that after DU145 cells were transfected with lncRNA H19 overexpression plasmid, the mRNA and protein profiles of E2F3 were dramatically heightened, but transfecting lncRNA H19 siRNA could reduce E2F3 expression. Moreover, E2F3 was under-expressed in miR-194 mimic-transfected cells as compared with the control group, whereas transfecting the miR-194 inhibitor could generate the opposite effect (Figure 4K–4R). All findings demonstrated that lncRNA H19 could positively up-regulate E2F3 expression via miR-194.

### The influence of lncRNA H19 and miR-194 on prostate cancer cell proliferation, apoptosis, invasion, and migration

**Figure 5:**
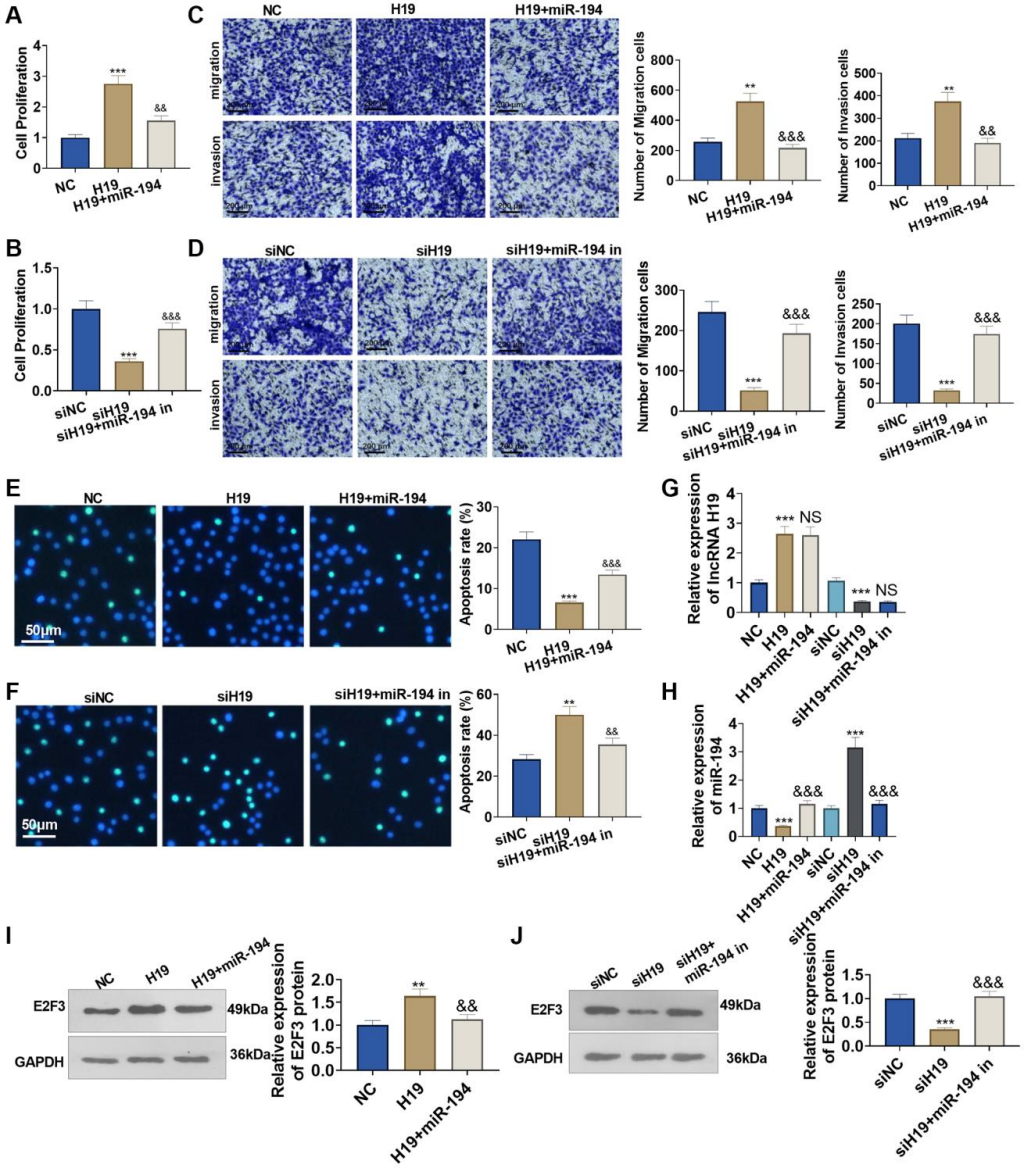
**The influence of lncRNA H19 and miR-194 on DU145 cell proliferation, apoptosis, invasion, and migration.** H19 overexpression plasmids and si-H19 were transfected into DU145 cells, and miR-194 mimics or inhibitors were administered for 24 hours culture. (**A**, **B**) CCK8 assay detected cell proliferation. (**C**, **D**) Transwell monitored cell migration and invasion. Scale bar = 100 μm. (**E**, **F**) TUNEL staining examined apoptosis. Scale bar = 50 μm. (**G**, **H**) qRT-PCR examined the profiles of lncRNA H19 and miR-194. (**I**, **J**) Western blot was used for examining E2F3 protein level. ^**^P < 0.01, ^***^P < 0.001 (vs. NC or siNC); ^&&^*P* < 0.01, ^&&&^*P* < 0.001 (vs. H19 or siH19), *n* = 3.

To understand the influence of lncRNA H19 and miR-194 on PCa cells, we administered miR-194 mimics or inhibitors after lncRNA H19 overexpression plasmids or lncRNA H19 siRNA was transfected. CCK8 assay displayed that the proliferation ability of lncRNA H19-transfected cells was increased compared with the control group, and the proliferation of cells co-transfected with lncRNA H19 and miR-194 mimics was decreased as compared with cells overexpressing lncRNA H19 (Figure 5A, 5B). Transwell assay indicated that the invasion cell count was elevated in the lncRNA H19-transfected group, but overexpression of miR-194 could counteract the promoting effect of lncRNA H19 on cell invasion (Figure 5C, 5D). TUNEL staining checked apoptosis, suggesting that the high profile of lncRNA H19 could suppress cell apoptosis, while such inhibitory effects could be eliminated by elevating miR-194 expression (Figure 5E, 5F). On the other hand, transfecting lncRNA H19 siRNA could repress DU145 cell proliferation, invasion, and migration and facilitate apoptosis, whereas miR-194 inhibitors might invert the regulatory function of lncRNA H19 siRNA (Figure 5A–5F). We detected lncRNA H19, miR-194, and E2F3 levels in DU145 cells. It was found that H19 overexpression repressed miR-194 level and promoted E2F3 mRNA and protein levels (Figure 5D–5J). Oppositely, H19 knockdown enhanced miR-194 level and decreased E2F3 levels. Moreover, miR-194 addition also attenuated E2F3 that was promoted by H19 overexpression (Figure 5G–5J). All discoveries unveiled that lncRNA H19 restrained miR-194 expression to bolster prostate cancer cell proliferation and migration.

### The influence of miR-194 and E2F3 on prostate cancer cell proliferation, apoptosis, invasion, and migration

**Figure 6:**
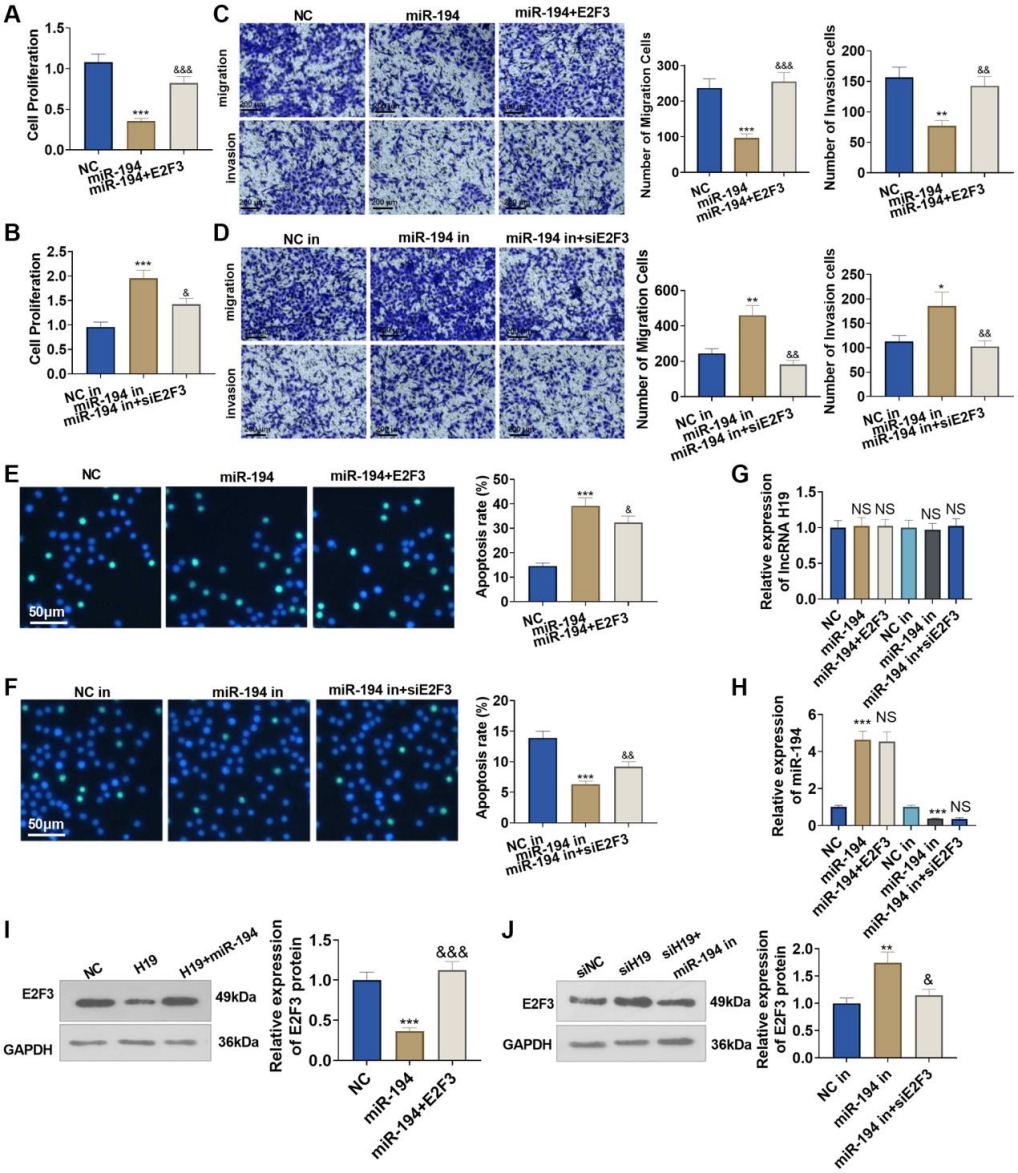
**The impact of miR-194 and E2F3 on DU145 cell proliferation, apoptosis, invasion, and migration.** E2F3 overexpression and si-E2F3 plasmids were transfected into DU145 cells for 24 hour culture after miR-194 mimics or inhibitors were administered. (**A**, **B**) CCK8 assay checked cell proliferation. (**C**, **D**) Transwell assay examined cell migration and invasion. Scale bar = 100 μm. (**E**, **F**) TUNEL staining detected cell apoptosis. Scale bar = 50 μm. (**G**, **H**) qRT-PCR examined the profiles of lncRNA H19 and miR-194. (**I**, **J**) Western blot was used for examining E2F3 protein level. ^***^P < 0.001 (vs. NC or NC-in); ^&^*P* < 0.05, ^&&^*P* < 0.01, ^&&&^*P* < 0.001 (vs. miR-149 or miR-149 in), *n* = 3.

To figure out the impact of miR-194 and E2F3 on prostate cancer cells, we transfected E2F3 overexpression plasmids or E2F3 siRNA after miR-194 mimics or inhibitors were added. As a result, transfecting miR-194 mimics could dampen DU145 cell proliferation, invasion, and migration and boost apoptosis, but the regulatory function of miR-194 was counteracted by E2F3 in cells co-transfected with miR-194 mimics and E2F3 (Figure 6A–6F). On the other hand, miR-194 inhibitors could enhance cell proliferation, invasion, and migration and suppress apoptosis, but transfecting E2F3 siRNA could upend the regulatory function of miR-194 inhibitors (Figure 6A–6F). We tested lncRNA H19, miR-194, and E2F3 levels in DU145 cells. It was found that miR-194 overexpression repressed E2F3 level and had no significant effects on H19 expression (Figure 6G–6J). Oppositely, miR-194 inhibition increased the mRNA and protein level of E2F3, while it had no significant function on H19 expression n (Figure 6D–6J). Our findings exhibited that miR-194 played its part as an oncogene in prostate cancer, but E2F3 inverted the anti-tumor function of miR-194.

### Brevilin A curbed the pro-cancer function of lncRNA H19

**Figure 7:**
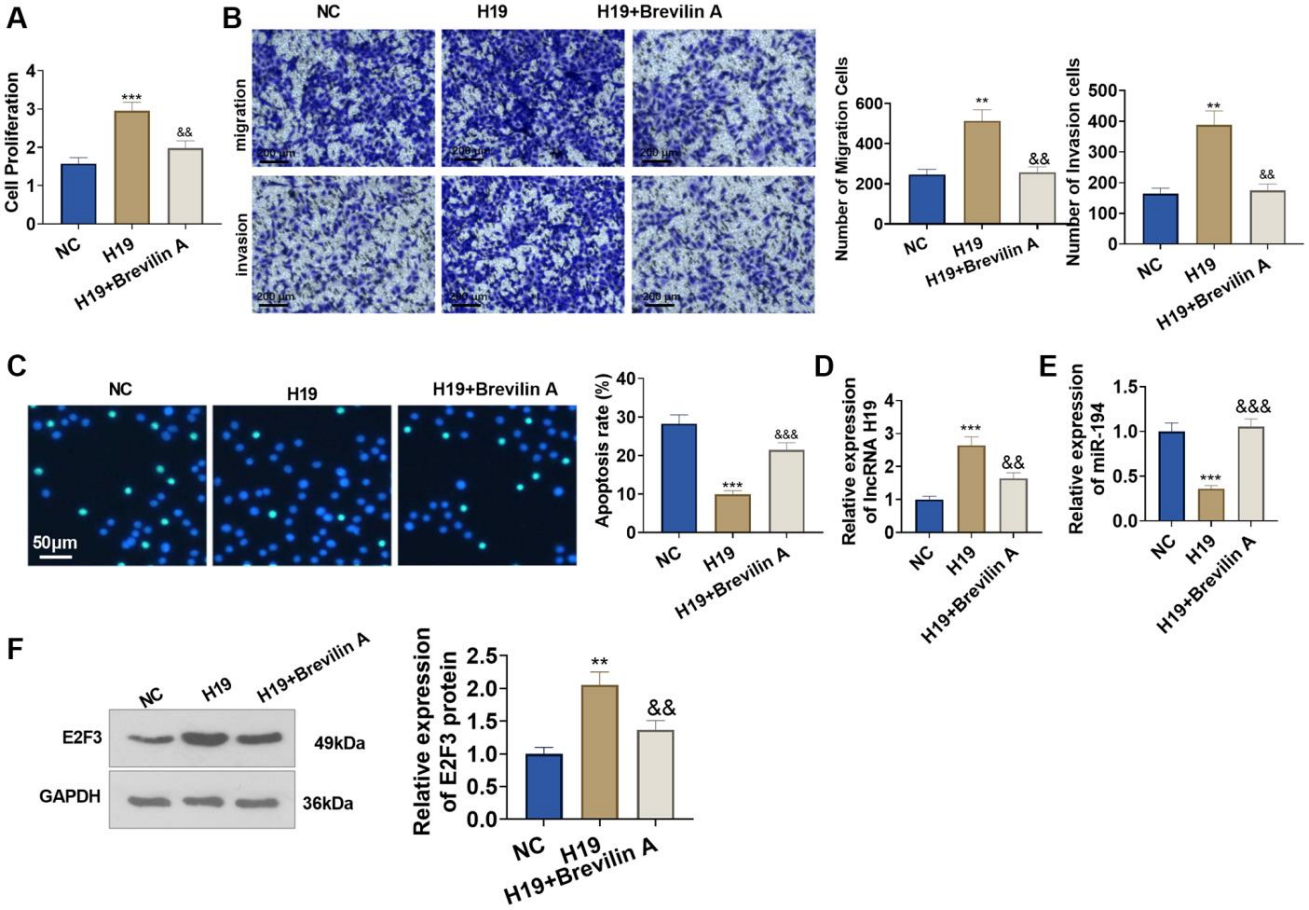
**Brevilin A hampered the pro-cancer function of lncRNA H19.** Brevilin A was utilized to treat DU145 cells stably transfected with lncRNA H19 overexpression plasmids. (**A**) CCK8 assay checked cell proliferation. (**B**) Transwell assay examined cell migration and invasion. Scale bar = 100 μm. (**C**) TUNEL staining detected cell apoptosis. Scale bar = 50 μm. (**D**, **E**) qRT-PCR examined the profiles of lncRNA H19 and miR-194. (**F**) Western blot was used for examining E2F3 protein level. ^**^P < 0.01, ^***^P < 0.001 (vs. NC); ^&^*P* < 0.05, ^&&^*P* < 0.01, ^&&&^*P* < 0.001 (vs. H19), *n* = 3.

To investigate the influence of Brevilin A on the pro-cancer function of lncRNA H19, we adopted Brevilin A (20 μM) to treat the cells transfected with lncRNA H19 overexpression plasmid for 24 hours. CCK8 assay displayed that in contrast with the H19 group, Brevilin A impeded prostate cancer cell viability (Figure 7A). Transwell assay denoted that by contrast to the H19 group, Brevilin A dampened prostate cancer cell migration and invasion (Figure 7B). TUNEL assay signified that as opposed to the H19 group, Brevilin A bolstered apoptosis (Figure 7C). Moreover, Brevilin A reduced E2F3 and H19 level, and enhanced miR-194 level (vs. H19 group, Figure 7D–7F). These discoveries revealed that Brevilin A curbed the pro-cancer function of lncRNA H19.

### The functions of Brevilin A and lncRNA H19 in the nude mouse xenograft model of prostate cancer

**Figure 8:**
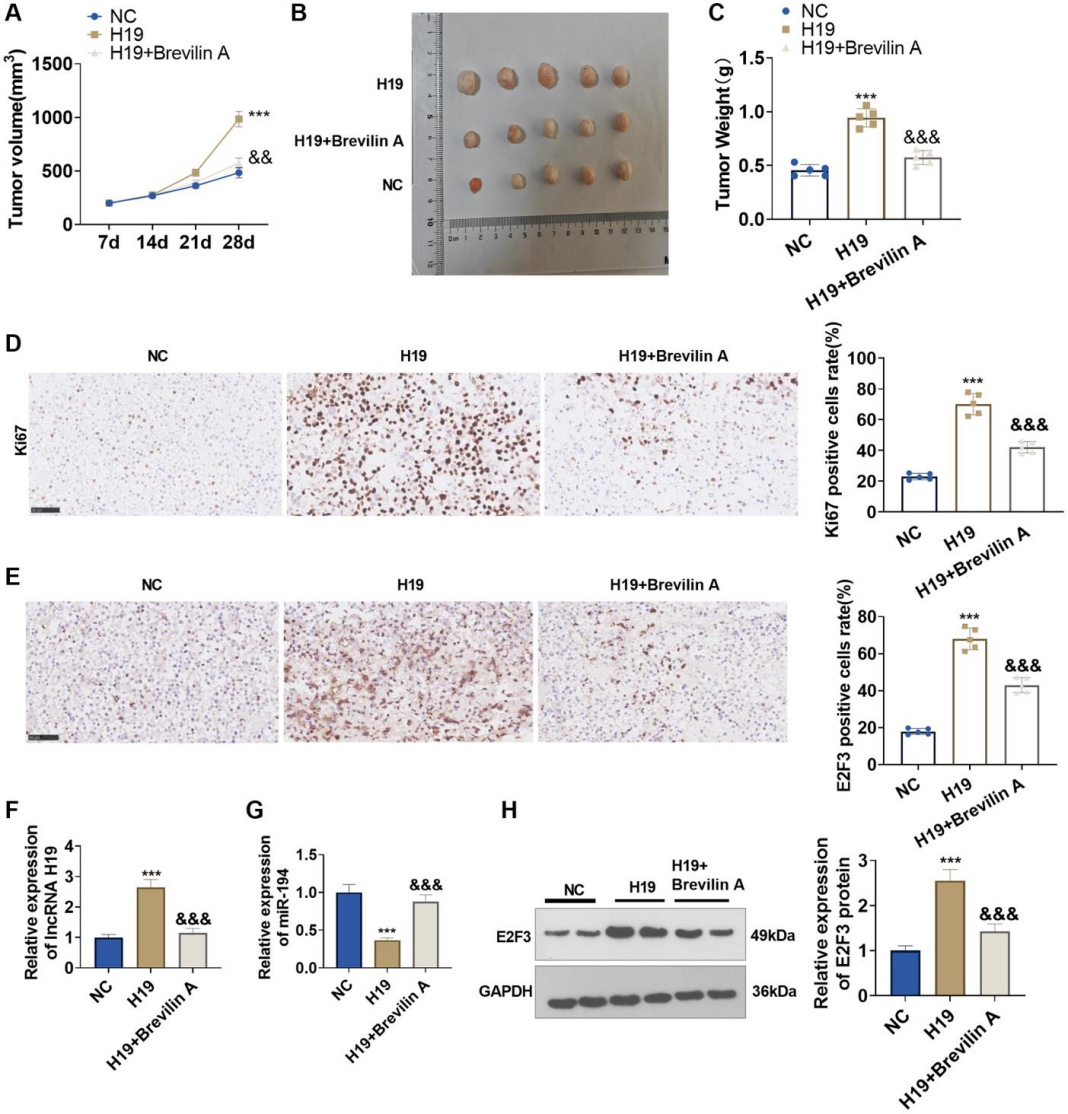
**The functions of Brevilin A and lncRNA H19 in the prostate cancer nude mouse xenograft model.** DU145 cells, stably transfected with lncRNA H19 overexpression plasmids, were taken to construct a nude mouse xenograft model of prostate cancer, and Brevilin A was harnessed for treatment. The tumors were excised 28 days later. (**A**) The tumor volume. (**B**) The images of tumors. (**C**) The tumor masses. (**D**, **E**) Immunohistochemistry determined Ki67 and E2F3 expressions. (**F**, **G**) qRT-PCR examined the profiles of lncRNA H19 and miR-194. (**H**) Western blot was used for examining E2F3 protein level. ^***^P < 0.001 (vs. NC); ^&&^*P* < 0.01, ^&&&^*P* < 0.001 (vs. H19), *n* = 5.

To dig into the functions and mechanisms of Brevilin A *in vivo*, we established a nude mouse xenograft model of prostate cancer using DU145 cells stably transfected with lncRNA H19 overexpression plasmids and adopted Brevilin A for treatment. Twenty-eight days later, the tumors were excised. We discovered that lncRNA H19 overexpression augmented the tumor volume and mass, whereas Brevilin A attenuated the tumor volume and mass (Figure 8A–8C). Immunohistochemistry signified that by contrast to the control group, the lncRNA H19 group witnessed an increase in Ki67 expression, while in contrast with the lncRNA H19 group, the lncRNA H19 +Brevilin A underwent a reduction in its expression (Figure 8D). Later on, we uncovered that as compared with the control group, the lncRNA H19 group had an uplift in E2F3 expression, whereas in contrast with the lncRNA H19 group, the lncRNA H19 +Brevilin A group had a decline in E2F3 expression (Figure 8E). We conducted qRT-PCR for testing lncRNA H19, miR-194, and E2F3 levels in the formed tumor tissues. It was found that H19 upregulation led to H19 and E2F3 enhancement, but miR-194 was repressed. Brevilin A addition partly rescued miR-194 level and reduced H19 and E2F3 levels (Figure 8F–8H). These findings demonstrated that Brevilin A repressed lncRNA H19/miR-194/E2F3 expression to curb prostate cancer growth.

## DISCUSSION

Lately, Brevilin A has displayed various characteristics like anti-proliferation, anti-cell viability, as well as inducing cell cycle arrest and apoptosis in multiple cancer cells [24, 25]. It exerts its function as an anti-tumor bioactive molecule in breast cancer [26], gastric cancer [27], colorectal cancer (CRC) [28], and other cancers [5–7]. Here, we discovered that Brevilin A displayed an anti-tumor effect on prostate cancer as well. Our experiments denoted that Brevilin A could frustrate prostate cancer cell proliferation, migration, and invasion, repress lncRNA H19 expression, and invert the pro-cancer function of lncRNA H19.

In men, PCa has become the most prevalent malignant tumor outside of the skin. When it comes to metastatic castration-resistant prostate cancer, Docetaxel is frequently the first chemotherapy to demonstrate an increase in overall survival [29]. However, challenges also come in the chemotherapy of PCa, including (1) limited choice for individualized treatment approach for prostate cancer, (2) side effects including incontinence, impotence, erectile dysfunction, bowel problems, and hot flashes, (3) therapy resistance of many advanced prostate cancer cases, (4) recurrence, (5) personal and psychological impact. Overcoming these challenges requires a multifaceted approach involving early detection, comprehensive treatment plans, effective management of side effects, and ongoing support from healthcare professionals, caregivers, and support groups [30–34]. Active agents or molecules from natural products are regarded as a novel approach for antitumor adjuvant therapy [35]. Several active compounds from *Centipeda minima* have been revealed with antitumor functions. For example, Arnicolide D promotes mitochondrial membrane damage, activated apoptosis pathway, and induced ferroptosis of breast cancer cells [36]. Thymol and carvacrol are two chemical compositions of essential oil from *Centipeda minima* (EOCM) [37], and they both show antitumor activities [38, 39]. Here, we also showed that Brevilin A can prevent cell proliferation, migration, invasion and induce apoptosis, which has been confirmed in other tumor cells [5–7, 26–28].

LncRNAs are known to be implicated in multiple biological processes and modulate protein-coding genes at many levels, including epigenetic regulation, transcriptional and post-transcription regulation [40, 41]. A number of studies have revealed that lncRNAs can play an essential role in the initiation and development of prostate cancer [42]. For instance, up-regulating PlncRNA-1 can dampen the apoptosis of prostate cancer cells and enhance proliferation [43]. Up-regulation of lncRNA PCAT14 can impede tumor cell proliferation [44]. In addition, previous studies have indicated that lncRNA H19 is abnormally expressed in multiple tumors like ovarian cancer [45], bladder cancer [46], and lung cancer [47] as a proto-oncogene [48]. Our study demonstrated that lncRNA H19 exhibited a higher level in prostate cancer tissues compared to adjacent normal tissues. *In-vitro* assays also denoted that lncRNA H19 overexpression could facilitate PCa cell proliferation, invasion, and migration and suppress apoptosis, whereas lncRNA H19 down-expression could produce the opposite phenomenon, showing that lncRNA H19 could exert a promotive function in prostate cancer. Previous studies have shown that signal transducer and activator of transcription 3 (STAT3) is a vital transcription factor that undergoes phosphorylation and translocates to the nucleus where it binds to DNA and regulates gene expression. The genes regulated by STAT3 include those involved in cell proliferation, angiogenesis, and immune response [49]. STAT3 can access the active promoter region of *H19* to upregulate H19 expression [50]. Brevilin A functions as an inhibitor of Stat3 pathway [51]. Therefore, we guessed whether Brevilin A has a role in H19 expression. Interestingly, we found that Brevilin A significantly attenuated the expression of H19 and dampened the oncogenic functions of H19 in PCa.

CeRNAs are known to regulate gene expression via competitively binding to miRNAs, and lncRNAs can sponge a variety of miRNAs to curb their expression and elicit gene silence [52]. Here, TargetScan discovered that the 3′-UTR of lncRNA H19 had a binding site for miR-194, and dual-luciferase reporter gene assay confirmed that lncRNA H19 and miR-194 had a targeted correlation. miR-194 is a small, non-coding RNA that plays a critical role in regulating gene expression in various cellular processes, including cell proliferation, differentiation, and apoptosis [53]. Several studies have shown that miR-194 dysregulation may play a role in cancer development and progression. In breast cancer, miR-194 has been shown to be downregulated, and this downregulation is associated with tumor proliferation, migration, and invasion. In addition, miR-194 inhibits tumor growth and metastasis in the HER2-positive breast cancer [54]. In hepatocellular carcinoma (HCC), miR-194 suppresses tumor cell stemness and enhances tumor cell’s sensitivity to sorafenib [55]. In bladder cancer, miR-194 subsequently inhibits the malignant phenotypes of cancer cells through repressing the Wnt/β-catenin signaling pathway [56]. In the present study, miR-194 expression was vigorously attenuated in prostate cancer cells and tissues, indicating that changes in miR-194 expression were possibly associated with prostate cancer. *In-vitro* experiments reflected that miR-194 curbed DU145 cell proliferation, invasion, and migration and bolstered apoptosis. Additionally, Brevilin A significantly promoted miR-194 those data suggested that miR-194 is a tumor-suppressive gene in PCa.

Further experiments showed that miR-194 reversed the regulatory impact of lncRNA H19 on PCa cell biological behaviors, which signified that miR-194 was a key regulatory target of lncRNA H19. Interestingly, previous studies have confirmed that H19 aggravates diseases progression by targeting and inhibiting miR-194 expression. For example, elevated H19 in pancreatic ductal adenocarcinoma can accelerate cell proliferation and migration of the tumor cells by negatively regulating miR-194 [57]. Moreover, the H19-miR-194 axis is also involved in the epithelial-mesenchymal transition of colorectal adenocarcinoma [58], gallbladder cancer cell proliferation [59], 5-Fu resistance in colorectal cancer [60], suggesting that the H19-miR-194 axis is essential in tumor development. miRNAs could exert their functions via regulating downstream target genes, which can suppress target gene translation by complementary pairing with the 3′-UTR of mRNAs [61]. Here, we firstly predicted that miR-194 and E2F3 had a potential binding site and verified that miR-194 could specifically combine with the 3′-UTR of E2F3. Afterwards, our further findings signified that after the transfection of lncRNA H19 or miR-194 inhibitors in DU145 cells, E2F3 expression was dramatically elevated, but lncRNA H19 siRNA or miR-194 mimics could restrain E2F3 expression. The above findings revealed that lncRNA H19 could positively modulate E2F3 expression via targeting miR-194.

Current studies have displayed that multiple cytokines can contribute to cell cycle regulation, and the transcription factor E2F family is one of the important regulation links [62]. As a member of the E2F family, E2F3 is known to be an essential cytokine in progression through G1 and into the S-phase of the cell cycle. E2F3 and cyclin D1 participate in cell cycle regulation and DNA replication, which generates a link with anti-oncogenes and oncogenes [63]. Researches have indicated that the aberrant profile of E2F3 protein was correlated with tumorigenesis [64, 65]. In bladder cancer, E2F3 is inextricably related to its initiation, and overexpression of E2F3 can culminate in tumorigenesis [66]. Rb inactivation can cause a rise in E2F expression, which boosts prostate cancer development [67]. Here, E2F3 mRNA and protein expressions exhibited a considerable increase in prostate cancer cells and tissues, indicating that the abnormal profile of E2F3 had a potential correlation with prostate cancer. Furthermore, high E2F3 expression could reverse the regulatory effects of miR-194 mimics on DU145 cells, while E2F3 inhibition could eliminate the regulatory function of miR-194 inhibitors, suggesting that miR-194 could modulate prostate cancer cells via targeting E2F3. Through the establishment of a prostate cancer nude mouse model, we discovered that lncRNA H19 bolstered tumor growth, augmented positive Ki67 cells, and enhanced E2F3 expression, whereas Brevilin A dampened the promoting effect of lncRNA H19. These findings demonstrated that Brevilin A could restrain lncRNA H19 expression to modulate miR-194/E2F3 activation and frustrate prostate cancer growth.

In conclusion, our study has confirmed that Brevilin A impedes prostate cancer cell proliferation, migration, and invasion and exerts its anti-tumor function in prostate cancer through modulating the lncRNA H19/miR-194/E2F3 signaling pathway. Notwithstanding, whether Brevilin A can be of great help for prostate cancer treatment in the future still awaits further investigation.
